# George Orwell, objectivity, and the reality behind illusions

**DOI:** 10.1177/03010066221094756

**Published:** 2022-05-16

**Authors:** David Rose

**Affiliations:** School of Psychology, 3660 University of Surrey, Guildford, UK

**Keywords:** illusions, reality, objectivity, truth, veridicality, philosophy of perception, metaphysics

## Abstract

Illusions are commonly defined as departures of our percepts from the veridical representation of objective, common-sense reality. However, it has been claimed recently that this definition lacks validity, for example, on the grounds that external reality cannot possibly be represented truly by our sensory systems, and indeed may even be a fiction. Here, I first demonstrate how novelist George Orwell warned that such denials of objective reality are dangerous mistakes, in that they can lead to the suppression and even the atrophy of independent thought and critical evaluation. Second, anti-realists assume their opponents hold a fully reductionist metaphysics, in which fundamental physics describes the only ground truth, thereby placing it beyond direct human sensory observation. In contrast, I point to a more recent and commonly used alternative, non-reductive metaphysics. This ascribes real existence to many levels of dynamic systems of information, emerging progressively from the subatomic to the biological, psychological, social, and ecological. Within such a worldview the notion of objective reality is valid, it comes in part within the range of our senses, and thus a definition of illusions as kinds of deviations from veridical perception becomes possible again.

*Reality is that which, when you stop believing in it, doesn't go away*.

— Philip K. Dick

## Illusions and Objective Reality

There has been much debate recently over the nature of perceptual illusions, which are perhaps most commonly defined as deviations from veridical perception. But there have been several recent attacks on this idea, some of which amount to the suggestion that illusions do not exist at all. (For brief introductions to the opinions on both sides, see Maniatis, 2015, Rose, 2018a, and Shapiro & Hedjar, 2019. More extensive discussions are in Hoffman, 2019; Rogers, 2019; and Todorović, 2020; and in the collections edited by Hickok, 2015; Reeves & Pinna, 2017; and Shapiro & Todorović, 2017.)

In brief, there are at least four lines of criticism. One argues that illusions are so many and various they have nothing in common that links them into a single category of percepts. The second denies we can make any distinction between illusory and ‘normal’ everyday perception, for example, because they share the same mechanisms. Third is the denial that normal perception is veridical, because *all* our percepts are inevitably biased or imperfect in some way, if to varying degrees. Fourth is the claim that veridical perception is impossible in principle. In this paper, I address the fourth of these arguments. It arises from the presumption either that (a) there is no objective (*mind-independent*) external reality to perceive, and thus no factual ground truth about it, or (b) although there is an objective reality we cannot perceive it because it is entirely beyond the range of our senses.^1^

Similarly, Todorović (2020) has recently discussed the denials that illusions can be defined as discrepancies between what we perceive and what the external world is like ‘in reality’. Although he concedes that definition is too broad, nevertheless he agrees that illusions fit within the category (‘illusions are indeed discrepancies from reality, but not all discrepancies from reality are illusions’: p. 1133). Thus their existence still stands or falls with the existence of an objective reality or a factual ground truth that acts as the standard for our perceptual apparatus to match (with as high a degree of veridicality or truth-likeness as possible) and without which there can be no contrast between veridical and illusory percepts.

However, in his iconoclastic Editorial in *Perception* satirising the notion of an ‘all seeing eye’, Koenderink (2014, p. 2) has denied the very possibility of objectivity:

“The All Seeing Eye belief neatly ties in with naive notions of objectivity. ‘Objective facts’ are states of affairs as seen by the All Seeing Eye. The exploits of scientists are aimed at views that approximate the All Seeing Eye's view as closely as possible. This is what science is about. … Although you are in the best of company if you fall for this silly story, the All Seeing Eye is a *delusion*.”

For phenomenologically oriented thinkers such as Koenderink, there is no objective and independent reality: they believe a sensory or perceptual component is necessary for existence—a how-things-seem or appear to a viewer or sensor. Thus the perceptual category of ‘illusions’, defined as errors or mismatches between perception and the objective (mind-independent) truth or fact of ‘what is out there’ in reality, becomes void.

Now, in the background to this debate is a long dispute between realism and anti-realism in the History of Ideas, running from Plato right up to the present day (for reviews, see: McMahon, 2001; Braver, 2013; Pagden, 2013; Sebold, 2014; Wolin, 2019). Here, I will focus on the depiction by George Orwell^2^ of how anti-realist attitudes can manifest themselves in culture and politics—with dire consequences upon the individual. In promoting the denial of objective reality and truth, these philosophies actually suppress a person's ability to see and think freely about their world. It is therefore suggested here that any similar denial of our common-sense understanding of objective reality in scientific research on perception and illusions should be resisted.

I begin by presenting George Orwell's criticism of anti-realism (next three sections). Then I describe the metaphysical foundation assumed by anti-realists in current research on perception. Finally, I point to one alternative metaphysics that is consistent with at least some forms of perceptual realism.

## Big Brother is Watching You

Perhaps Orwell's most famous work is his portrayal of a future dystopia in the novel *Nineteen Eighty-Four* (Orwell, 1949; Waddell, 2020). In that totalitarian nightmare, ubiquitous portraits of the political leader known as ‘Big Brother’, and prominent signs saying ‘Big Brother is Watching You’, remind citizens that their beloved dictator (or his secret police) have them under constant surveillance wherever they go, and even within their own homes:

“Even from the coin the eyes pursued you. On coins, on stamps, on the covers of books, on banners, on posters and on the wrapping of a cigarette packet — everywhere. Always the eyes watching you and the voice enveloping you. Asleep or awake, working or eating, indoors or out of doors, in the bath or in bed — no escape. Nothing was your own except for the few cubic centimetres inside your skull.”

Yet even those few cc's of privacy were penetrable by the regime:

“The hypnotic eyes gazed into his own. It was as though some huge force were pressing down upon you — something that penetrated inside your skull, battering against your brain, frightening you out of your beliefs, persuading you, almost, to deny the evidence of your senses.”

Orwell then continues to lay out the psychological consequences of being subjected to such continuous and intense coercive pressure:

“In the end the Party would announce that two and two made five, and you would have to believe it. It was inevitable that they should make that claim sooner or later: the logic of their position demanded it. Not merely the validity of experience, but the very existence of external reality, was tacitly denied by their philosophy. The heresy of heresies was common sense. And what was terrifying was not that they would kill you for thinking otherwise, but that they might be right. For, after all, how do we know that two and two make four? Or that the force of gravity works? Or that the past is unchangeable? If both the past and the external world exist only in the mind, and if the mind itself is controllable—what then?”

Note especially the sentence ‘Not merely the validity of experience, but *the very existence of external reality,* was tacitly denied by their philosophy’, as it reminds us of the most relevant point about Orwell's thesis: its significance for our current debate about whether illusions exist.

## Doublethink: Truth and Untruth About Reality

Now, Orwell fought in the Spanish Civil War, and briefly wrote propaganda for the British during the Second World War. After that, he was much exercised by the way that politicians distort the truth for their own ends—and sometimes even change it (e.g. by their rewriting of history, such as denying that a particular battle had occurred although they knew it had). However, the corruption consists not simply in telling outright lies, but also in deliberately telling contradictory ‘truths’, with the aim of shaking people out of their belief that there is any such thing as hard and fast ‘objective truth’ or ‘facts’ (Tinline, 2020).

It is worth reading some further passages from Orwell (1949) to get the subtlety of the techniques used in promoting both ‘doublethink’ and acceptance that what you think ‘true’ can change at any time if the politicians so wish it.

“Doublethink means the power of holding two contradictory beliefs in one's mind simultaneously, and accepting both of them. … To tell deliberate lies while genuinely believing in them, to forget any fact that has become inconvenient, and then, when it becomes necessary again, to draw it back from oblivion for just so long as it is needed, to deny the existence of objective reality and all the while to take account of the reality which one denies—all this is indispensably necessary. Even in using the word doublethink it is necessary to exercise doublethink. For by using the word one admits that one is tampering with reality; by a fresh act of doublethink one erases this knowledge; and so on indefinitely, with the lie always one leap ahead of the truth. …

If one is to rule, and to continue ruling, one must be able to dislocate the sense of reality.”

While torturing the novel's hero Winston Smith for the ‘thoughtcrime’ of not loving Big Brother, a Party official strengthens the brainwashing:

“Only the disciplined mind can see reality, Winston. You believe that reality is something objective, external, existing in its own right. You also believe that the nature of reality is self-evident. When you delude yourself into thinking that you see something, you assume that everyone else sees the same thing as you. But I tell you, Winston, that reality is not external. Reality exists in the human mind, and nowhere else. Not in the individual mind, which can make mistakes, and in any case soon perishes: only in the mind of the Party, which is collective and immortal. Whatever the Party holds to be truth, is truth. It is impossible to see reality except by looking through the eyes of the Party. …

We control matter because we control the mind. Reality is inside the skull. You will learn by degrees, Winston. There is nothing that we could not do. Invisibility, levitation—anything. I could float off this floor like a soap bubble if I wished to. I do not wish to, because the Party does not wish it. You must get rid of those nineteenth-century ideas about the laws of Nature. We make the laws of Nature.”

## Consequences of Doublethink

The Party official clearly espouses philosophies that have been expressed (in the real world) by the several dictators and philosophers of both Left and Right who inspired Orwell's characterisation of Big Brother and his regime (Dwan, 2018). These philosophies include relativism, constructivism and idealism. (Put briefly, relativism is the idea that objective truth does not exist, only people's opinions, which are all of equal validity—or at best truth is a consensus among people, even if it is one dictated by certain others, such as those in power at any given time. Constructivism is the notion that everything we believe, think and experience is determined purely by sociopolitical or linguistic influences.^3^ Idealism is disbelief in the reality of any external world, only in subjective experience.) This combination of ideas comprises a worldview that crystallised originally from various strands of anti-realist opinion among philosophers opposed to the Enlightenment. In the late 20th century, their ideas spread into the broad cultural movements of post-modernism and populism (originally via Foucault, Lyotard, Derrida, Latour, and others in France). Those belief systems in turn gave rise to the current era of ‘post-truth’ and *anti-science*, with proliferation and widespread dissemination of ‘alternative facts’ and ‘fake news’—and with consequential dramatic manifestations in recent politics (e.g. Nichols, 2017; Fuller, 2018; Kakutani, 2018; McIntyre 2018; Salgado, 2018; Hopf et al., 2019; Pomerantsev, 2019; Wolin, 2019; Pond, 2020; Zotzmann & Vassilev, 2020; Rauch, 2021; Sidky, 2021).

But as Orwell also makes clear, it is not simply that loss of belief in objectivity opens the way to the tyranny of those with the loudest voices (or the most authority, charisma, guns, or money—or the catchiest slogans). The true problem is far deeper and more subtle. Instead of clarity and consistency, the powerful deliberately say contradictory things. This leads to the complete discrediting of everything that everybody says, however powerful or well qualified as ‘experts’ they may be. As soon as you have concluded you have no certain, true, and reliable facts upon which to base decisions, then you cease even any attempt to make them—you give up trying to think for yourself. Your resulting state of lassitude and passivity clears the way for those in power to act as they please, without even minimal scrutiny or criticism from anyone else.

## Perception

In a striking echo of the processes Orwell so described, some recent commentators on perception (e.g. Maniatis, 2015; Rose & Brown, 2015; Gomez-Marin, 2020; see also Hickok, 2015) have suggested that inconsistent, confusing, or contradictory statements about reality also appear in the perception research literature. For example, they point out, various researchers have denied the mind-independent existence of material objects,^4^ and of the sun before human minds existed,^5^ or have claimed that only phenomenal experiences are real,^6^ while nevertheless maintaining that we evolved by natural selection—thus implicitly (and often explicitly) accepting that our ancestors were actual organisms, with actual sense organs,^7^ in an objective world replete with sunshine, rocks, and rival organisms. Similarly inconsistently, some have denied that we perceive external reality correctly, as it ‘really’ is, without explaining how they know that such a reality exists at all, or how they know it is not as we perceive it, given that they also claim perception is the only source of knowledge that we have.^8^

These are of course not malicious acts of doublethink. Certainly, the researchers concerned have worked hard to clarify their (ex)positions. The discussion is ongoing. But might the effects on readers, however unintentional, be the same as they are when politicians make contradictory statements? That is, uncertainty and apathy about what is real or true, or even antipathy towards the issue altogether. For example, in recent surveys on this topic (Rose, 2018b, 2019a), 4 out of 31 vision researchers said they didn't understand Koenderink’s (2014) point, while 4 more said they didn't care. One of the latter added that he or she ‘[did not] think much can be learned that is of practical use without wasting time arguing this point.’ Now, if such antipathy becomes widespread, decision-making might then be surrendered to whatever famous, charismatic or immediate source of influence is the most dominant, with passive and uncritical acceptance of whatever that authority has most recently declaimed or pontificated to be the truth.^9^ There would be no more independent thinking and critical appraisal of ideas in the field.

## Reality

But what is this ‘reality’ that we should believe in? This is the vexed problem of metaphysics. For example, Hoffman (2019; see also Hickok, 2015) has famously presented the metaphor of percepts (apparent objects) as akin to a set of icons on a computer desktop: “The purpose of the desktop interface is not to show you the ‘truth’ of the computer—where ‘truth,’ in this metaphor, refers to circuits, voltages and layers of software. … These icons are useful, in part, because they hide the complex truth about objective reality” (2019, pp. xii–xiii). Indeed, many scientists similarly believe reality is at root only describable by fundamental physics, not in everyday perceptual terms (e.g. ‘Only the particles of physics exist’: Herzog, 2021). In this they echo Eddington (1928) and Sellars (1962), who distinguished between the ‘manifest image’ of an object in perception (such as a table) and its ‘scientific image’ in knowledge as a collection of moving charges in space.^10^

One problem with any such (micro-)reductionist worldview is that it is not clear how far ‘down’ you have to go to reach ‘reality’. This is an old debate (e.g. Hüttemann, 2003; Schaffer, 2003; Montero, 2006). Is there actually a bedrock or foundational level that is the one true reality and above which all else is simply a construction from those most basic building blocks? Or, as other metaphysical theories postulate, might there be no end, just an infinite chain of reduction (at least as far as we can tell, i.e. the Planck length: Liang, 2020), and thus no reality at all?

In the alternative view outlined next, the building blocks are always (at least, above the Planck length) further decomposable, like a Russian doll (except with multiple mini-dolls rather than one within each doll). But unlike the reductive view, every such doll has its own validity as a part of reality. Thus, the term *‘non-reductive*’ is standardly applied to the worldview. This thesis is of wide applicability throughout the sciences and humanities, where it synthesises multiple local disciplinary viewpoints and supersedes the old reductionist assumption that only fundamental physics studies the true reality, and can explain causation. Thus we can agree with Schaffer (2003, pp. 512–513) that: ‘Mesons, molecules, minds, and mountains are in every sense ontologically equal.’ ^11^

While there is no space here to give a full review and justification of this newer non-reductive metaphysics, I will briefly present three relevant ideas, which I hope will be sufficient to give the gist.^12^ First is the idea that Nature consists of multiple *levels* of dynamic interacting *systems*, nested more or less *hierarchically* within one another. Systems *emerge* by spontaneous self-organisation of components interacting with each other. The behaviours of those components are now *constrained* within the new higher-level system they have formed. Moreover, these lower-level components are themselves systems, similarly emerged from the level below them. This process applies recursively so that ultimately there are many levels of reality, not just that of the most fundamental physical building blocks, if any. It is *all* these multiple levels that comprise the material of reality and should be described by any comprehensive theory—and that hold the explanatory resources for our accounts of perception.

Second, it is natural to ask what these systems and their components are actually made of, or how they are ‘realized’. Any name is arbitrary here (since under monism there is nothing to contrast it with),^13^ but some say ‘energy’ (e.g. Tyler, 2015; Pepperell, 2018) while others prefer ‘*information’* or *‘pattern’*; I will go with the latter.^14^ In brief, we then have: ‘reality is the totality of information’ (Floridi, 2011, p. xiii),^15^ ‘To be is to be a real pattern …’ (Ladyman & Ross, 2007, pp. 226, 233, 253, …) and ‘it's real patterns all the way down’ (Ladyman & Ross, 2007, p. 228). If there is a basic unit of the universe, it is one bit of information: same or different, uniform or patterned, symmetrical^16^ or broken.

Thus, putting ideas one and two together, we have: reality is composed of multiple levels of ontologically real, hierarchically nested systems, which are patterns of emergent self-organising information. A given system's coming into interaction with another means the creation of a channel by which information transfers between them (Ladyman & Ross, 2007, pp. 307–309; Beni, 2020). If the transfer is bidirectional, they now form a higher-level system, which persists because its free energy or entropy^17^ is lower than that of all its previously separate progenitors. Its emergent properties comprise newly created information^18^ and *constrain* the potential behaviour (degrees of freedom) of its component parts, including their ability to separate or disconnect (Wilson, 2010; Blachowicz, 2013; Marshall et al., 2018; Albantakis et al., 2019; Rose, 2021). The same principle can generate level upon level, supersystem upon supersystems. Then, as Ladyman & Ross (2007, p. 300) put it: ‘Prices, neurons, peptides, gold, and Napoleon are all real patterns, existing in the same unqualified sense as quarks, bosons, and the weak force.’

Third, real existence is intimately linked with *causal* power (e.g. Chakravartty, 2007; Beni, 2020).^19^ This applies to social structures as much as to psychological, biological, or physical ones (Elder-Vass, 2010, 2012; Bennett, 2013; Lawson, 2013; Mingers, 2014; Abell & Engel, 2018). For example, nation states, not just their individual leaders, make war or peace with each other, which affects the futures of those entire nations as wholes. Companies engage in legally binding contracts with other companies. Government fiscal policy affects market behaviour and macroeconomic performance. The social ethos, Zeitgeist, social norms, and mores guide and direct the course and successfulness of whole societies and their philosophies. Thus, just as objects such as coronaviruses, umbrellas, and professors are real, so too are other higher-level emerged entities such as concepts, memes, reputations, invisible colleges, data sets, theories, and the laws of copyright.^20^ These are all the effects of causes and have causal effects on the world.

In sum, within this metaphysical picture, perception is causal information transfer (see also Lombardi & López, 2018; Godfrey-Smith, 2020).^21^

## Implications

So it is necessary to believe there are objective facts and truths about agreements and agriculture, elections and emotions, morals and murders, preferences and prejudice—otherwise we would have no standards against which to judge whether our words are truly meaningful, our actions genuinely effective or ethical, and our decisions and beliefs actually correct. Similarly, we need to believe there are objective facts and truths about portraits and parallel lines, stairs and spears, tigers and tables, ziggurats and zigzags, which we can use as a basis for our decisions on how and when to act—and against which we can judge whether our percepts are illusory or veridical.

Could we be post-modern relativists about perception, but still believe there are objective facts about society, politics, law, and history? How about vice versa? Wouldn't either bivalent stance be inconsistent, and thus make us guilty of doublethink, or at least give that appearance? Shouldn't our philosophies be at least consistent on all these issues?

In other words, if you believe that people are born and people die, that millions of people were killed in what is commonly known as the Holocaust, that theft is illegal in your country, that most people have two hands, that diamonds are denser than air, that the capital of France is called Paris, that vaccines protect us against viruses, … then you (at least implicitly) believe in objective reality.^22^

So, as such a realist, you should believe there can be perceptual illusions (as they are commonly defined, i.e. deviations from veridical perception). Although there are multiple levels of reality, and hence many ways reality can be described (Todorović, 2020, pp. 1174–1178), one can stipulate or specify which are the *relevant* ones for measuring the ground truth that perception should match, and that give the criteria for distinguishing the veridical from the illusory. As Todorović (2020, p. 1191) puts it: ‘… although multiple descriptions of reality may exist, once a particular description is chosen, well-defined criteria of correctness and error can be defined.’ For example, when asked to judge the length of a shaft in the Müller-Lyer figure, the relevant level is not that of the atoms or quarks, nor of molecules of ink, nor whether the line is in the fins-in or fins-out context, nor whether it depicts one edge of a building or room. It is the length of the line at the level where it can be measured, for example with a ruler on the piece of paper where it has been drawn. If you doubt the reality of that measurement then you may be confusing different levels, or trying to make a judgment across different levels. Illusions, like perception itself, must be defined with respect to a specified level of reality.

## Conclusion

Orwell has made us aware of the falsehoods, inconsistencies and tricks played on us by unscrupulous politicians. Denial of objective reality in perception research could lead to conceptual problems analogous to those caused by the denial of objective reality in wider culture and world affairs. Believing one denial without the other would be inconsistent. Hence belief in objective reality by perception researchers, and thus in the possibility (if not the uncontroversially defined existence) of perceptual illusions, is both justified and mandatory.

## Notes

1. Claim (a) can arise within continental philosophy and phenomenology, because they make no distinction between external reality's existence (ontology) and our knowledge of it (epistemology). Claim (b) may then be proposed to avoid the pitfalls of idealism immanent in claim (a) (e.g. Sebold, 2014; Sparrow, 2014; Rose, 2021). Surveys of scientific researchers (Rose, 2018b, 2019a) have also found some that say veridical perception is impossible because our senses are too limited—though it is not clear whether they think reality lies entirely beyond those limits or only in part (i.e. that ‘veridical’ implies complete). However the understanding of veridicality I will follow here is that it applies only to what is within the range of our senses.

2. Eric Blair (1903–1950), commonly known as George Orwell, was a British essayist, critic, and journalist. He also wrote some of the most influential novels of the 20th century, commenting particularly on issues in politics and society.

3. Unlike relativism, constructivism allows that beliefs might reflect an objective truth by chance. Unlike constructivism, relativism allows that beliefs might arise at random or idiosyncratically. In common usage, however, both philosophies co-refer to the social construction of internal belief systems.

4. For example, ‘Here, we show that under the assumption of a mind independent world of physical particles there is no space for a mind *in*dependent world of objects. … If there is one fundamental ontology (fundamental physics), one cannot have any other ontology (objects) in addition.’ (Herzog & Doerig, 2021, p. 1). A second example is in Hoffman’s book *The Case Against Reality* (2019, p. xii) where he states: ’our perceptions of snakes and apples, and even of space and time, do not reveal objective reality.’ Taken together with the title, this clearly suggests there are no such objects, no such reality. Yet a few sentences later he says‘… the very language of objects in space and time is the wrong language to describe objective reality.…our perceptions mislead us about objective reality …,. So there is an objective reality, despite the book’s title! It was perception’s ability to *reveal* that he was denying in the first sentence above, not the existence of objective reality itself. In addition, like Herzog & Doerig (2021) and others, Hoffman denies physical objecthood. However, given that there is a reality out there, it cannot be a point singularity, nor can it be perfectly homogeneous, isotropic, or symmetrical along all its dimensions or parameters, whatever they may be (not space and time, according to Hoffman). No, reality must be structured or ‘*clumpy*’ in some way (e.g. Millikan, 2017; Morganti, 2021). But those entities, ripples, patterns, or clusters deserve the name ‘objects’, in that they are more or less distinct parts of and events in reality. Whether their boundaries are sharp or fuzzy, and how they are individuated, re-identified, and tracked by an observer (Millikan, 2017), are matters orthogonal to the metaphysics of the underlying reality. So a language of ‘objects’ is appropriate, even if as Hoffman claims they are not in reality the way they appear to us to be.

5. In dispute with Francis Crick, Hoffman (2019, p. xiv) first says: ‘In a series of letters that Crick and I exchanged a decade before his death, he argues that our perceptions match reality, and that the sun existed before anyone saw it.’ Later (pp. 42–44) he seems to accept that Crick was referring to the sun as an ‘unknowable’ Kantian noumenon, and then concludes (p. 45): ‘He [Crick] assumed that our ideas-of-things truly describe the thing-in-itself, so that the same vocabulary describes both. I rejected this assumption as implausible. But Crick thought it applied even to objects, space, and time.’ Indeed Meillassoux (2006), Woodward (2012), Vrahimis (2013), Sebold (2014, pp. 1–3), and Wiltsche (2016) have already used the ‘ancestrality of the sun’ example in critical discussions of phenomenology and its anti-realism. (Another example one might similarly consider is: did your parents exist before you were born?)

6. ‘Awareness simply is. It is synonymous with reality.’ (Koenderink & van Doorn, 2013, p. 14).

7. Are one's own sense organs real objects? If not, and they are just as unknowable as any other object, how can we possibly ever come to understand how perception works (which is after all our aim)—or what the word ‘perception’ even means? We are left with pure mysticism or brute, inexplicable phenomena.

8. How science knows what reality is like is a matter that modern philosophy of science now has a much better handle on (e.g. Chakravartty, 2007; Bschir et al., 2019) compared to the old empiricist assumptions still all too widely held in the scientific community—and in continental philosophers’ caricature of science as reductive ‘scientism’. Non-sensory processes are also at play in science, such as logic and reasoning, assessing ideas as to their degrees of plausibility, reliability, robustness and coherence with other ideas (as summarised in Rose & Brown, 2015, §§3–4).

9. Without belief in objectivity, aren't the editors and referees of scientific papers, or the organisers of symposia, free to assert or impose whatever point of view they wish, without even *trying* to be impartial, fair and unbiased in their assessments?

10. Eddington (1928, p. x) claimed he had two tables, an ordinary everyday one with colour and substantiality, and a scientific one which is ‘mostly emptiness’ containing ‘numerous electric charges rushing about with great speed.’ In reply, Ladyman & Ross (2007, p. 253) say that ‘… both the scientific image and the common-sense image can track real patterns.’ Is there any consistent relationship between reality and Hoffman’s desktop icons (even if the icons are arbitrary symbols)? There must be, else they would not be useful. But how is the causality of the link established? At some level of objective reality, a connection must form from physical reality to an icon. Therefore the icon is real too, else there would be a category error between them (the non-real percept and the objective physical reality) and Hoffman would have all the problems Descartes and Kant had in justifying their dualisms (as well as the obvious one that the ‘you’, the consciousness, who inspects and uses the desktop interface is blatantly a man-in-the-head). See also Elder-Vass (2012, pp. 246–248) and Rose (2019b).

11. A colourful metaphor for a reality in which everything can be reduced to a single, flat level is ‘desert ontology’ (Quine, 1960), whereas that with a rich jungle of entities growing at higher levels is ‘rainforest realism’ (Ross, 2000; see also Ladyman & Ross, 2007; Wimsatt, 2007).

12. I have of course had to synthesise and summarise across many sources here. Several general surveys are available in the literature (e.g. Capra & Luisi 2014; Gillett, 2016; Humphreys, 2016; Walloch, 2016; DiFrisco, 2017; Glennan & Illari, 2018; Gibb et al., 2019) as well as more discipline-specific sources pertaining to biology (Kesić, 2016; Noble et al., 2019; Brooks et al., 2021), physics (Hartmann & Valente, 2011; Glick et al., 2020), consciousness (Rose, 2006, 2012; Atmanspacher, 2015; Tyler, 2015), neuroscience (Pennartz, 2015; Boone & Piccinini, 2016), cognition (Fabry, 2018; Ramstead et al. 2021; Sims, 2021), and social and economic theory (Elder-Vass, 2012; Mingers, 2014; Lee et al., 2019; Pond, 2020).

13. Hoffman (2019, ch. 10) calls basic reality ‘consciousness’ or a network of conscious agents. While I have some sympathy with panpsychism (Skrbina, 2009; Alter & Nagasawa, 2015; Brüntrup & Jaskolla, 2017; Seager, 2020; Rose, 2021), the word ‘consciousness’ in this context has to be so qualified that it can be very misleading to apply it to this level. It may make sense to say one bit of information can become quantum-entangled with another (or comprises such an entanglement), but this is very far from being an explanation of consciousness-as-we-know-it.

14. ‘Energy’ and ‘information’ are actually interchangeable (e.g. Landauer, 1996; Collell & Fauquet, 2015; Parrondo et al., 2015; Street, 2016).

15. One leading contender in modern metaphysics is ‘structural realism’. It postulates that mathematics can give veridical descriptions of reality; conversely, reality is the structures or patterns described by the mathematical equations. Whether there is anything else beyond is open to debate (e.g. Chakravartty, 2007). Without going into these alternatives, I am here presenting ‘informational structural realism', in which the structures or patterns are information (see also Rose, 2021). Thus: “… the ultimate nature of reality is informational … mind-independent and constituted by structural objects that are neither substantial nor material … but cohering clusters of data … [in the sense of] differences de re, i.e. mind-independent, concrete, relational points of lack of uniformity…In short, Being and Information conventuntur [are interconvertible].” (Floridi, 2011, p. 340).
“… the basic idea behind [Informational Structural Realism] is quite simple: we are [informational organisms] dealing with black-boxes inside a grey-box. … these qualifications are [level of analysis]- dependent, in the same way as the distinction between being a system and being a component or unit of a system is. A black-box may be opened, but opening it transforms it into a grey-box, in which more black-boxes may be found. Whether ad infinitum we simply cannot say. It might be Russian dolls (informational objects) all the way in.” (Floridi, 2011, p. 371).

16. The referent here is mathematical symmetry and its disruption in the creation of pattern (Moss Brender, 2013; Rose, 2021).

17. The relation between free energy, entropy, and negentropy is complex and beyond the scope of this paper; but note the ‘free energy principle’ (Friston et al., 2006; Colombo & Wright, 2021; see also Brown & Friston, 2012, on its application to illusions) is currently the subject of some controversy (Biehl et al., 2021; Kirchhoff et al., 2021; Aguilera et al., 2022).

18. ‘Information’ (pattern, distribution) here refers not to the number of bits transmitted but to the semantic content of a message (Lombardi et al., 2015, 2016; Mingers & Standing, 2018; Ball et al., 2020; Godfrey-Smith, 2020). The receiver/perceiver must already contain a set of possible meanings (priors), whose number is to be reduced by the message, or whose probability distribution needs to be unflattened (made more spiky or uneven). Thus in meaningful interactions, each system creates, selects, or imposes a particular form/pattern or reduces the range of possible forms the other system can take. Conversely random noise inputs or ‘*fake news*', especially in abundance, make the probability distribution flatter and/or wider and thus less useful for making decisions.

19. Note that the notion of causation that applies in the systems worldview is more subtle and complex than in traditional reductionist billiards-ball physics. This is first because there can be multiple inputs to a system at work simultaneously and persistently, as well as diachronic feedback of effects to their sources. Second, following Heraclitus, change is spontaneous not forced. Within a level, change is dynamic flow, constrained by other such processes that ‘cause’ the wrong changes *not* to happen (Dobson & Rose, 1985; Nicholson & Dupré, 2018). In all, the term ‘causation’ (Aristotelian efficient), if used at all, is best restricted to interactions within a given level, while so-called top-down and bottom-up causation are better referred to as constraint and emergence, respectively (cf. Emmeche et al., 2000; Glennan, 2010; Rose, 2012; Gillett, 2016; Noble et al., 2019).

20. But note this does not mean their reification: they are not all ‘things’, but systems of information. They are processes rather than, or as well as, substances (Elder-Vass, 2010, p. 196; DiFrisco, 2017; Nicholson & Dupré, 2018; Meincke, 2019; Austin, 2020).

21. But whether you want to call *all* information transfer, causation or interaction, at any level of reality, ‘perception’ depends how narrowly you judge we should use the word ‘perception’. Different people may use this word to relate only (i) to phenomenal experience, (ii) to the identification or recognition of what we encounter, or (iii) more widely as metaphor for any knowledge-generating interaction (even via language: Millikan, 2017) within any level of reality (Rose, 2021). For example, Reeves & Dresp-Langley (2017) have shown how disputes arise between those who define perception in terms of appearance as opposed to veridicality. Additionally, Hoffman (2019) contrasts perception in terms of inverse optics as opposed to the guiding of ‘adaptive behaviour’. Allowing for such individual differences in stance (‘perspectivism’) may enable us to explain and perhaps reconcile the conflict between realist and relativist worldviews, according to Giere (2006); see also Chakravartty (2017), Berenstain (2020), Massimi & McCoy (2020) and Glick (2021). So, at any given level, different systems perceiving the same transmitting system may be changed in different ways that depend on the perceivers’ various pre-existing states. For example, the visual experiences of different people (or species) viewing the same object may differ. But those effects are constrained to be in some ways similar (not arbitrary or random) by the objective reality of the shared transmitting system or object.

22. This reasoning is of course in addition to standard justifications for objective realism, such as common sense (Reeves & Dresp-Langley, 2017; Peels et al., 2020; Peels & van Woudenberg, 2021) and the successfulness of science (e.g. Chakravartty, 2007; Rose & Brown, 2015; Bshir et al., 2019). Reality is far more than just a single and mysterious fundamental level. Even continental philosophy has recently (re)turned to the ontological reality of objects (e.g. Meillassoux, 2006; Ferraris, 2015; Gabriel, 2015; DeLanda, 2016; Harman, 2018).
